# The Mutual Relations of the Medical Profession and Its Press

**Published:** 1872-02

**Authors:** 


### EDITORIAL AND MISCELLANEOUS.

### THE MUTUAL RELATIONS OF THE MEDICAL PROFESSION AND ITS PRESS.

From an interesting and instructive address delivered by Dr. Horatio R. Storer, of Boston, (Editor of the Gynæcological Journal), before the annual meeting of the Association of Medical Editors, at San Francisco, May, 1871—of which body he was President—we extract the following, and desire to commend it to the medical profession of the South:

"In this connection, I would say one word concerning the relations that we hold to these patrons, our brethren of the profession itself. We have our work to do for them, and we all of us endeavor to do it well. They encourage us by their contributions to our pages, by kind messages in the letters they write to us, and, to an ever-increasing extent, by the money enclosures therein contained. And yet, though personally I have had every reason to be grateful upon each of the scores I have named, I am sure that you will agree with me when I say that the medical profession, as yet, falls far short of its duty towards its Press. Of the great number of practicing physicians in the United States, there is good reason to believe that but a comparatively small number subscribe for more than a single medical journal, and that a very, very great many take none at all. This is far from being as it should be. There is no other such means of keeping the busy practitioner afloat with the ever-swelling tide of discovery and improvement in practice as that which you, the periodical Press, afford. There is no other such solace for his weariness, rest for his busy brain. There are honorable exceptions, it is true, to the remark that I have made. The magazine under my own direction has a subscriber who wrote that the Gynæcological Journal was the *thirteenth* medical periodical that regularly came to his table; and this was a hard-working, over-driven physician, in a sparsely-settled country district, with no leisure for study, it would seem, than that afforded while in the saddle upon his daily beat; and yet I will venture to say that this gentleman, by this means, kept himself better informed, more completely at a level with the prominent men of the day, than thousands of city practitioners, with greater wealth, more leisure, infinitely more pretensions, and far less liberality towards the members of this Association.

"There is not a physician in this country, I dare affirm, who would not each year obtain his money back again, at compound interest, were he to subscribe for, and read with the most ordinary appreciation, a copy of each of the journals that we represent. One-half of the sum that most men throw away at auction sales for stale and musty editions of authors now far behind the age, expended in subscriptions for the medical journals of the day, would not only do much for the continued education of our friends in practice, and keep their minds alive to the improvements in methods of study and treatment constantly being made, but it would tend, infinitely, towards a greater appreciation of and respect for our own native medical writers, who, through the channels of communication you offer them, are becoming recognized, as never before, by the profession of foreign lands."

